# Supplementary material for: Long term but not short term exposure to obesity related microbiota promotes host insulin resistance
Source: Nat Commun. 2018 Nov 8;9:4681. doi: 10.1038/s41467-018-07146-5 (PMC6224578; doi:10.1038/s41467-018-07146-5)
Supplement: Supplementary file 1 — Supplementary Information [file 41467_2018_7146_MOESM1_ESM.pdf]

# Long term but not short term exposure to obesity related microbiota promotes host insulin resistance

**Foley et al.**

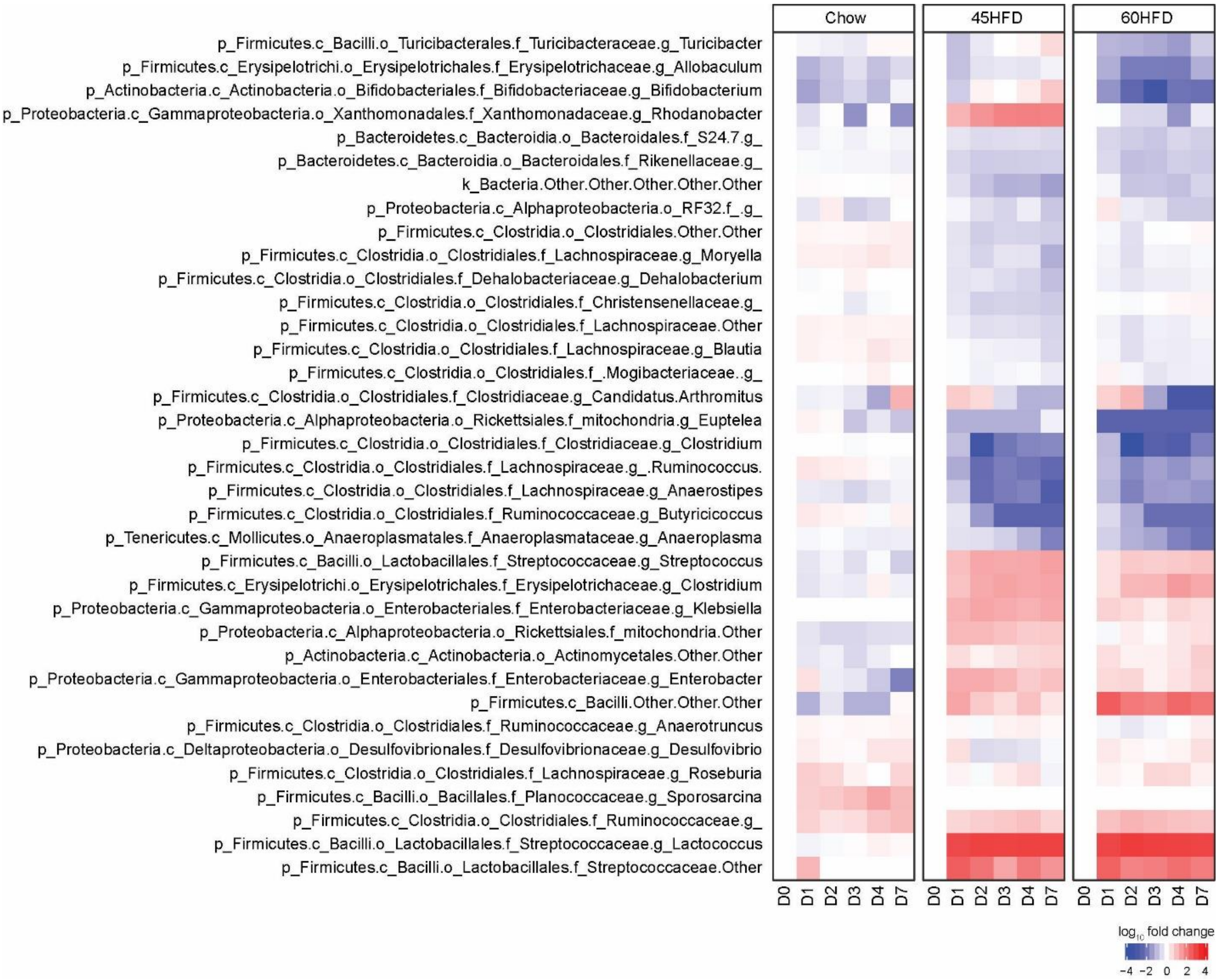


**Supplementary Figure 1:** Stability of early changes in the microbiota during high fat feeding. Time courses are shown for changes in abundance of taxa that were significantly different between Chow, 45% HFD, and 60% HFD on day 3. Non-parametric analysis of variance for each taxon between groups was conducted using the Kruskal-Wallis test. Taxa that passed the significance threshold of $p < 0.05$ were analyzed using the pairwise Wilcoxonon rank sum test. Correction for multiple hypothesis testing (FDR) was calculated using the Benjamini-Hochberg method. Statistical significance was accepted at $p < 0.05$. For the heatmap, the relative abundance for each taxon was normalized to Day 0 within each diet group and $\log_{10}$-transformed. The time courses for the relative abundance changes for each significant taxon (without normalization to Day 0) are shown below. Values represent the mean ± SEM (N=7-8).

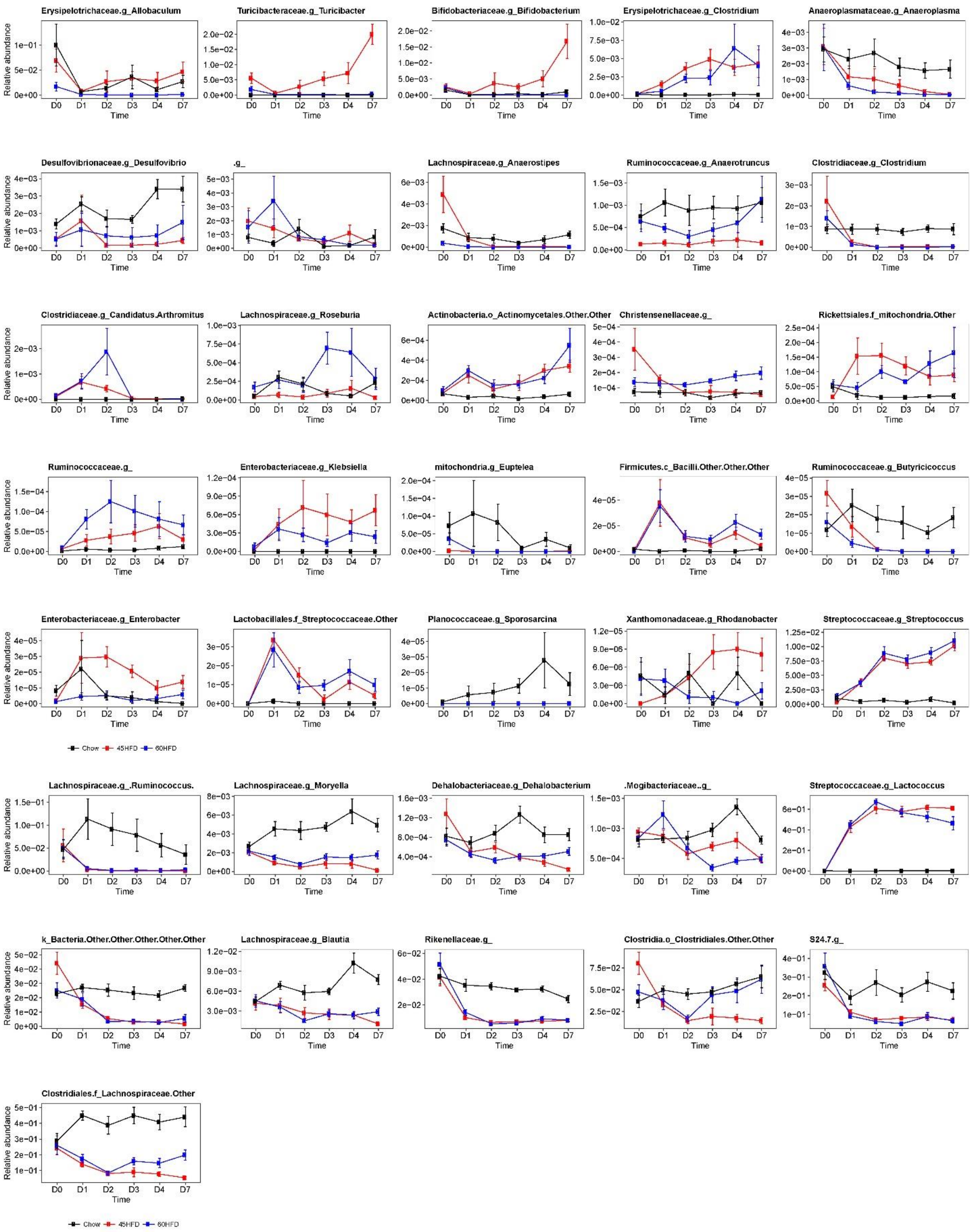


**Supplementary Figure 1-continued**

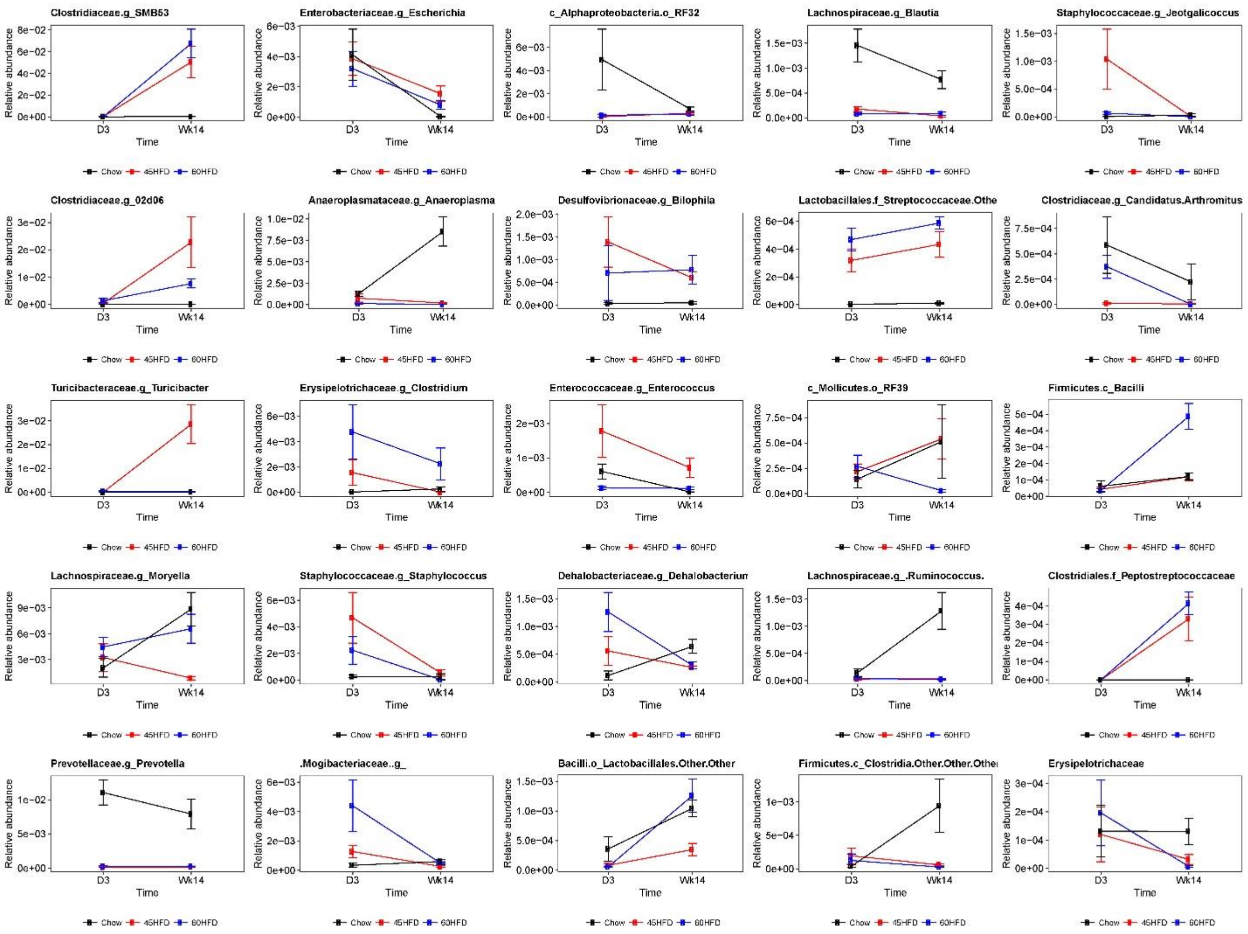


**Supplementary Figure 2:** Relative abundances of taxa at Day 3 versus Week 14 of high fat feeding. The same taxa are shown in Figure 3. These taxa were significantly changed ($p < 0.05$) in the high fat fed mice (45% or 60% HFD) compared with the Chow fed mice on either day 3 or week 14. For each time point (Day 3 or Week 14), non-parametric analysis of variance for each taxon between the diet groups was conducted using the Kruskal-Wallis test. Taxa that passed the significance threshold of $p < 0.05$ were analyzed using the pairwise Wilcoxonon rank sum test. Correction for multiple hypothesis testing (FDR) was calculated using the Benjamini-Hochberg method. Statistical significance was accepted at $p < 0.05$. The time courses for the relative abundance changes for each significant taxon (without normalization to Chow diet) are shown. Values represent the mean ± SEM (N=12-13).

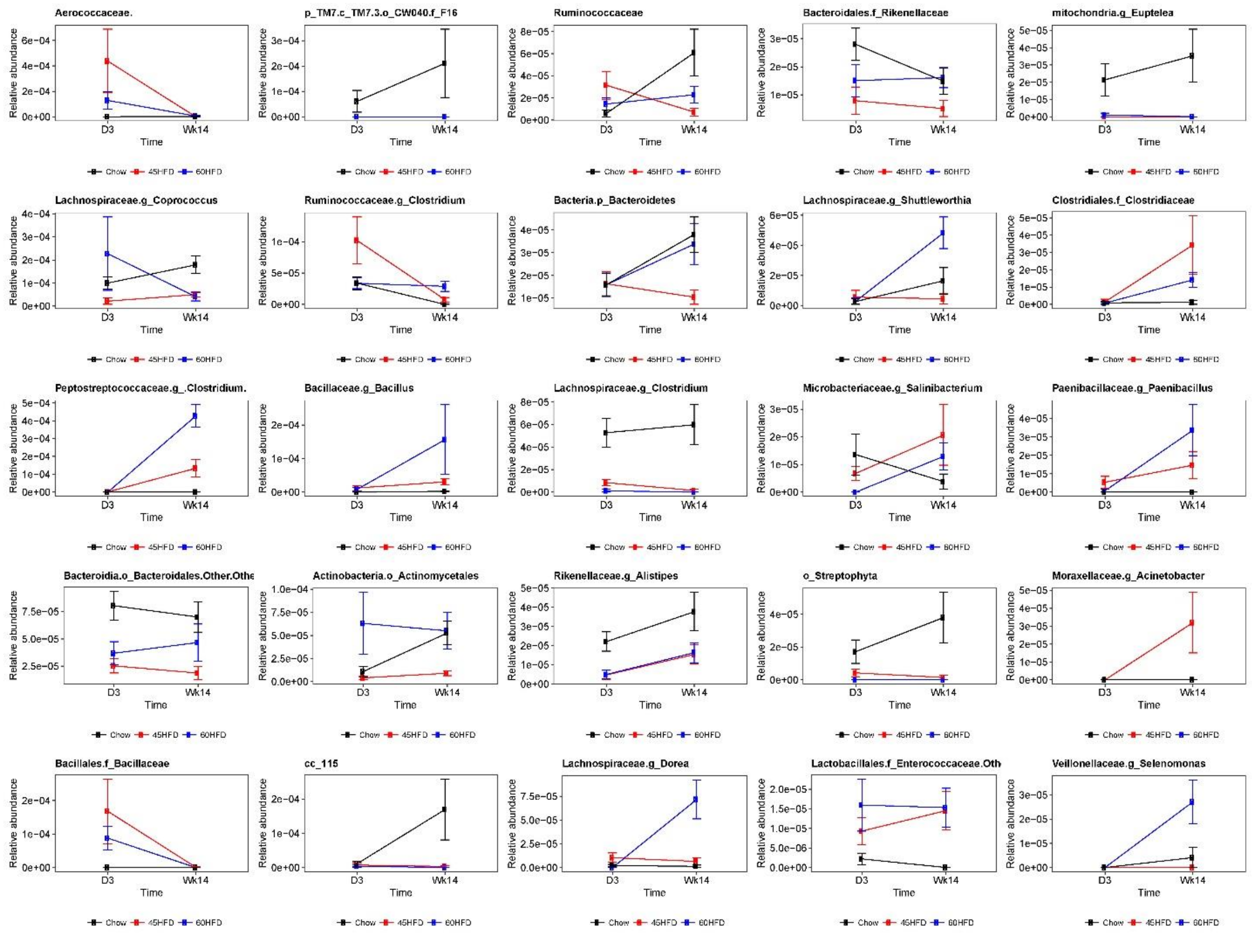


**Supplementary Figure 2 - continued**

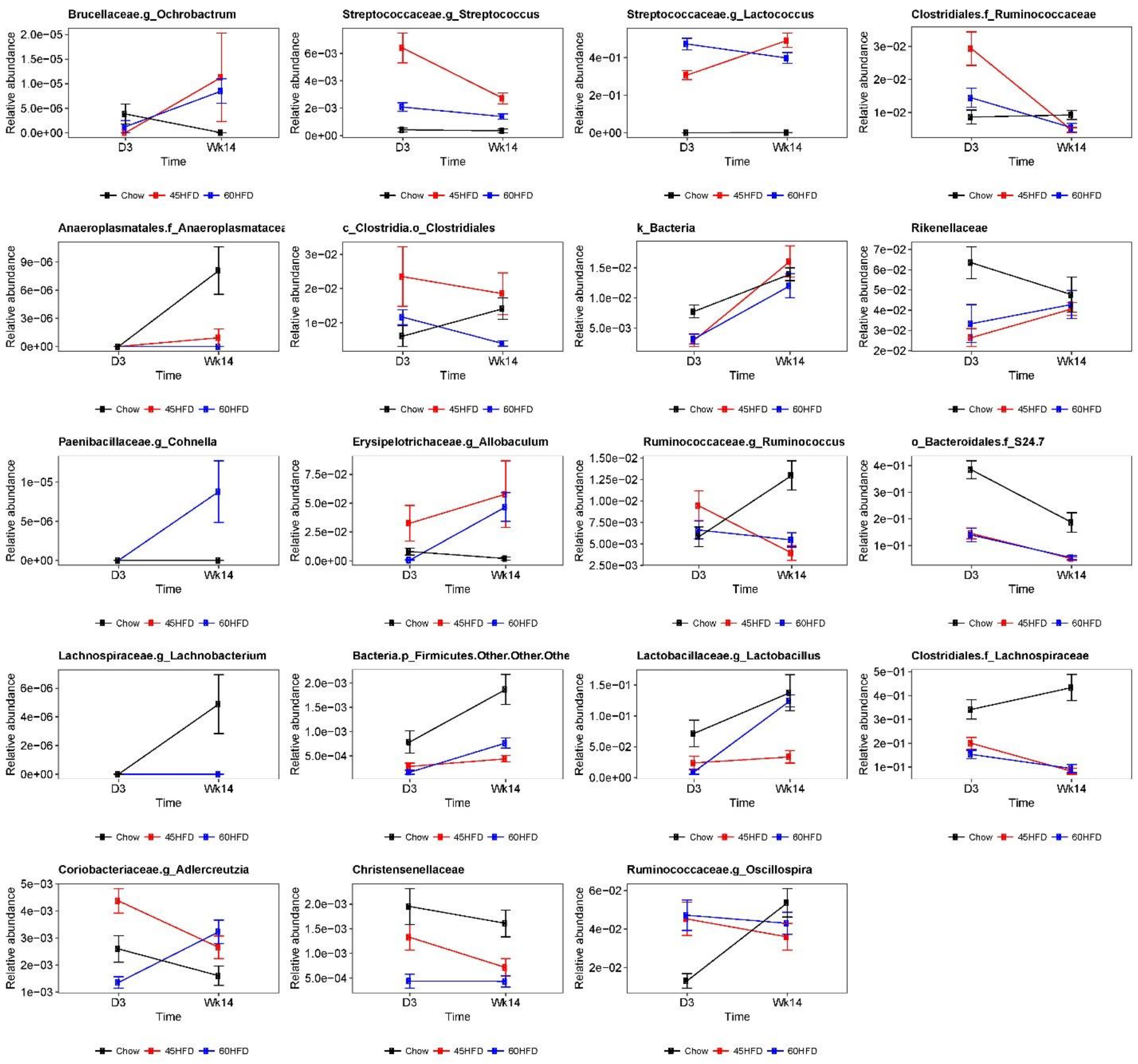


**Supplementary Figure 2 - continued**

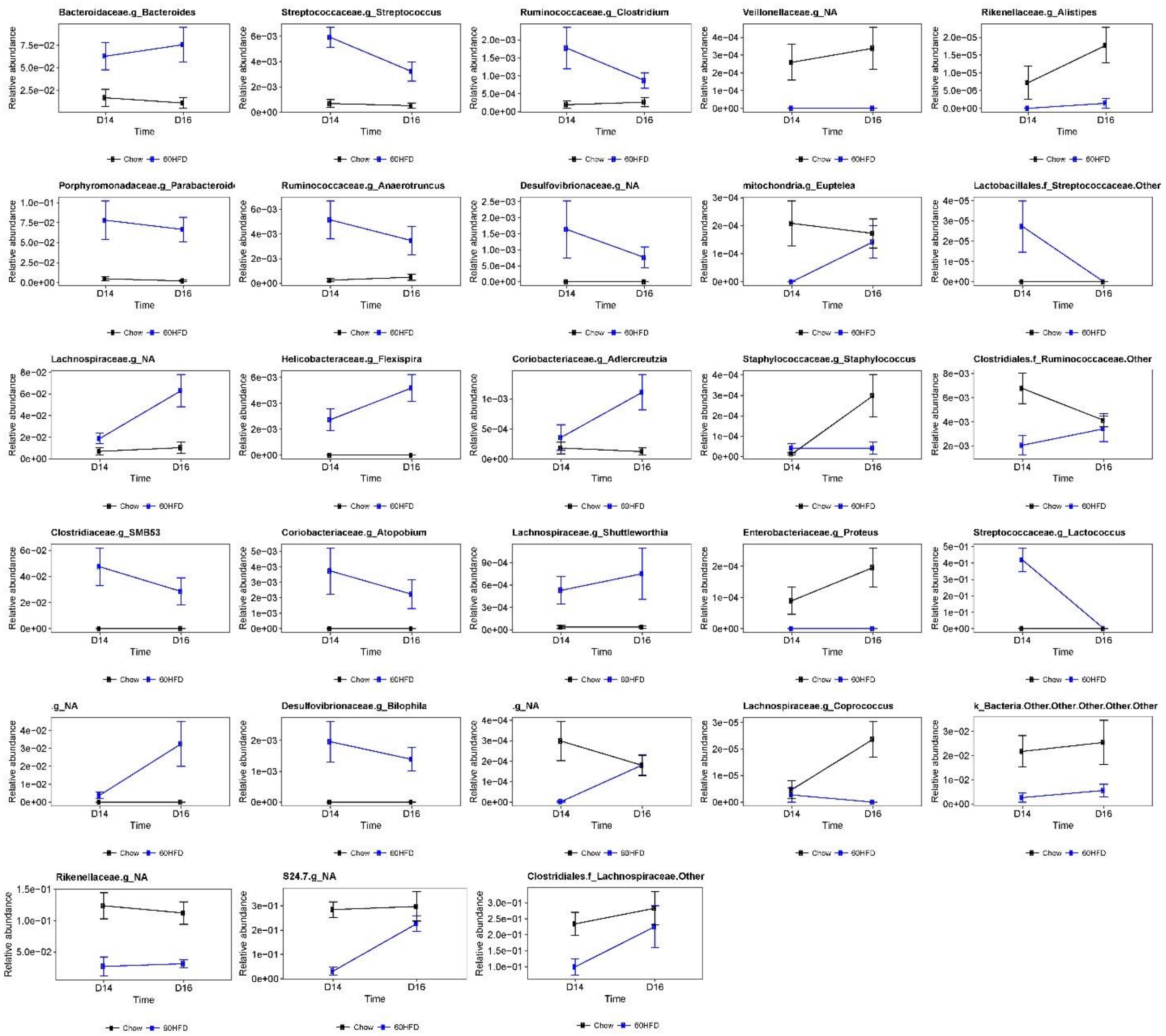


**Supplementary Figure 3:** Relative abundances of taxa during short term high fat feeding and diet removal. The same taxa are shown in Figure 4. These taxa were significantly changed ($p < 0.05$) in the different treatment groups compared with the Chow Day 14. Non-parametric analysis of variance for each taxon between the treatment groups (Chow day 14, Chow Day 16, 60% HFD Day 14, 60% HFD Day 16) was conducted using the Kruskal-Wallis test. Taxa that passed the significance threshold of $p < 0.05$ were analyzed using the pairwise Wilcoxonon rank sum test. Correction for multiple hypothesis testing (FDR) was calculated using the Benjamini-Hochberg method. Statistical significance was accepted at $p < 0.05$. The time courses for the relative abundance changes for each significant taxon (without normalization to Chow Day 14) are shown. Values represent the mean ± SEM (N=8).
